# Response of Juvenile *Saccharina japonica* to the Combined Stressors of Elevated pCO_2_ and Excess Copper

**DOI:** 10.3390/plants12051140

**Published:** 2023-03-02

**Authors:** Wenze Zhang, Lianghua He, Jiangqi Pan, Yuhong Zhou, Ruxiang Ge, Sufang Li, Yunyun Shi, Xinhua Chen, Yaoyao Chu

**Affiliations:** 1 College of Marine Sciences, Fujian Agriculture and Forestry University, Fuzhou 350002, China; 2 Key Laboratory of Marine Biotechnology of Fujian Province, Institute of Oceanology, Fujian Agriculture and Forestry University, Fuzhou 350002, China; 3 Department of Aquaculture and Aquatic Sciences, Kunsan National University, Gunsan 54150, Republic of Korea; 4 Laboratoire Génie des Procédés et Matériaux (LGPM), CentraleSupélec, Université Paris-Saclay, 91190 Gif-sur-Yvette, France

**Keywords:** biochemical compositions, copper, growth, ocean acidification, photosynthetic characteristics, *Saccharina japonica*

## Abstract

Coastal macroalgae may be subjected to global and local environmental stressors, such as ocean acidification and heavy-metal pollution. We investigated the growth, photosynthetic characteristics, and biochemical compositions of juvenile sporophytes of *Saccharina japonica* cultivated at two pCO_2_ levels (400 and 1000 ppmv) and four copper concentrations (natural seawater, control; 0.2 μM, low level; 0.5 μM, medium level; and 1 μM, high level) to better understand how macroalgae respond to ongoing environmental changes. The results showed that the responses of juvenile *S. japonica* to copper concentrations depended on the pCO_2_ level. Under the 400 ppmv condition, medium and high copper concentrations significantly decreased the relative growth rate (RGR) and non-photochemical quenching (NPQ) but increased the relative electron transfer rate (rETR) and chlorophyll *a* (Chl *a*), chlorophyll *c* (Chl *c*), carotenoid (Car), and soluble carbohydrate contents. At 1000 ppmv, however, none of the parameters had significant differences between the different copper concentrations. Our data suggest that excess copper may inhibit the growth of juvenile sporophytes of *S. japonica*, but this negative effect could be alleviated by CO_2_-induced ocean acidification.

## 1. Introduction

Copper is an essential micronutrient for algae, as it can be involved in many metabolic processes, such as electron transport in photosynthesis and enzymatic reactions in which it acts as a cofactor [1,2]. Total copper concentrations in natural seawater vary around the world but have been reported to be less than 1 μg/L [2,3]. However, as a result of industrial activities and wastewater emissions, the concentration of this metal in coastal seawater has increased several-fold, exceeding the tolerance capability of some seaweeds [4,5,6]. As a result, it has been widely reported that excessive copper inhibited algal growth and photosynthesis, as well as reduced chlorophyll content [6,7,8]. For example, the growth rates of *Ulva compressa* [3], *Ulva reticulata* [9], and *Porphyra haitanensis* [10] were significantly reduced when algae were subjected to high copper concentrations. Toxic effects of copper on the photosynthetic activities of macroalgae, such as *Gracilaria domingensis* [11], *Ulva prolifera* [12], *Ecklonia cava*, and *Ulva pertusa* [13], have been recorded. In these studies, decreases in the maximum quantum yield (*F_v_/F_m_*), maximum electron transport rate (rETR_max_), and pigment content were also found.

Since the industrial revolution, the oceans have absorbed approximately one-third of atmospheric CO_2_ emitted to the atmosphere, resulting in a drop in seawater pH, a phenomenon known as ocean acidification (OA) [14,15]. The growing atmospheric CO_2_ concentration is anticipated to be between 800 and 1000 ppm by the end of this century [14]. Numerous studies have found that CO_2_-induced OA affects algal photosynthetic activity, which in turn affects growth [16,17,18]. However, because of the difference in inorganic carbon acquisition mechanisms, macroalgae respond to OA in species-specific ways [16,18,19,20]. The majority of macroalgae use HCO_3_^−^ and/or CO_2_ in photosynthesis via carbon concentration mechanisms (CCMs) [21], while a few species only use CO_2_ via passive diffusion [22,23]. The latter does not require additional energy for carbon acquisition, implying that OA may benefit these species by lowering the energy costs of photosynthesis [24]. As such, OA can display divergent impacts on macroalgae (i.e., negative, neutral, and positive).

Macroalgae in coastal oceans may face a simultaneous shift in several environmental drivers, including heavy-metal pollution and ocean acidification. The simultaneous action of ocean acidification and heavy-metal exposure may have complex and diverse consequences [25,26]. Furthermore, because OH^−^ and CO_3_^2−^ complex with metal ions in seawater and decreased pH results in lower OH^−^ and CO_3_^2−^, OA affects their bioavailability and toxicity by increasing heavy metals’ solubility [27,28,29,30]. Copper pollution is a common form of heavy-metal pollution in coastal areas. Several studies on the coupled effects of OA and copper pollution on macroalgae have recently been published [6,12,29], and the combined effects may differ from the individual effects of each stressor, showing synergistic or antagonistic effects. For example, an increased pCO_2_ level reduced the inhibition rate of copper on the growth of *U. prolifera* [12] and weakened its inhibition of the maximum photosynthetic efficiency (*F_v_/F_m_*) of *Sargassum fusiforme* [6]. In contrast, copper and OA synergistically reduced the growth and photosynthetic performance of *Ulva linza* [29]. These various responses reflect specific-species capabilities. However, to date, few experiments have been conducted to evaluate the potential combined impacts of OA and copper on kelp species.

*Saccharina japonica*, a brown seaweed, is a foundational species in subtidal and intertidal habitats in the northwestern Pacific region, including China, Japan, and Korea [31,32]. It not only has significant ecological effects on marine ecosystems by providing habitats, shelters, and breeding areas for a variety of marine organisms [33] but also has significant economic value, such as providing food and raw materials for chemical products, pharmaceuticals, and cosmetics [34,35]. The individual effects of OA and copper on *S. japonica* have been studied [19,36,37]. These studies revealed that OA could significantly alter meiospore germination, fecundity, the reproductive success of the microscopic stage, and the growth and photosynthetic physiology of sporophytes of *S. japonica* [19,36,37,38]. Furthermore, investigations regarding the effect of copper on *S. japonica* showed a reduction in the development of female gametophytes and the growth of young sporophytes [39]. These results indicated that the growth and physiology of the microscopic stage or sporophytes of *S. japonica* would be significantly affected by OA or copper. Nevertheless, it is not well known how juvenile sporophytes of *S. japonica* respond to OA and copper in isolation and combination.

Therefore, in this study, we cultured juvenile sporophytes of *S. japonica* with four levels of copper (natural seawater; 0.2 μM; 0.5 μM; and 1 μM) and two levels of pCO_2_ (400 and 1000 ppmv). The aim was to investigate the combined effect of OA and copper on the growth, photosynthetic performance, and biochemical composition of *S. japonica* and illustrate how OA and excess copper affect the physiology of this alga.

## 2. Results

### 2.1. Carbonate Parameters

The effects of pCO_2_ and copper conditions on the seawater carbonate parameters were determined (Table 1). A two-way ANOVA analysis (*p* = 0.05) showed that pCO_2_ had a significant effect on all parameters except for total alkalinity (TA), whereas copper concentrations had no significant effect on any of the parameters. The post hoc Duncan comparison (*p* = 0.05) showed that, compared to 400 ppmv, the average pH and CO_3_^2−^ at the 1000 ppmv level decreased from 7.87–7.90 to 7.53–7.60 and from 80.1–84.8 μmol kg^−1^ to 38.5–44.7 μmol kg^−1^ at the four copper concentrations, respectively. However, HCO_3_^−^ and DIC increased from 2020.9–2029.0 μmol kg^−1^ to 2105.6–2128.2 μmol kg^−1^ and from 2132–2138 μmol kg^−1^ to 2205–2232 μmol kg^−1^, respectively.

### 2.2. Relative Growth Rates

**Figure 1:**
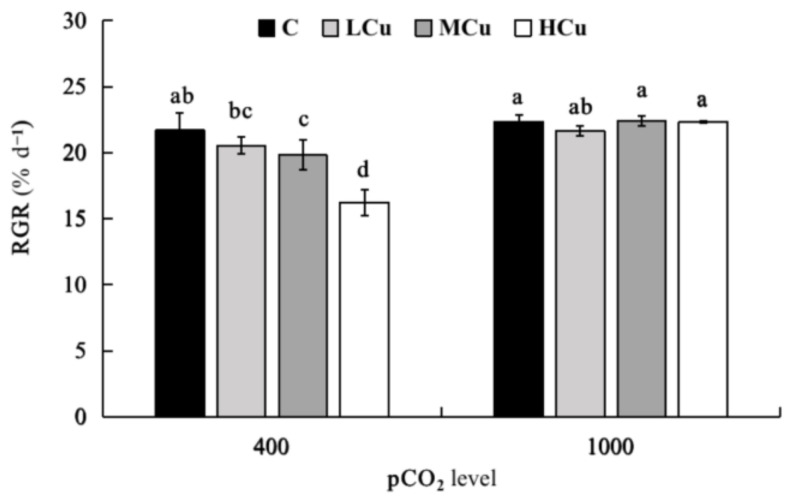
Relative growth rates of *S. japonica* grown under different pCO_2_ levels and copper concentrations. C (control, natural seawater); Lcu (low copper concentration, 0.2 μM); Mcu (medium copper concentration, 0.5 μM); Hcu (high copper concentration, 0.2 μM). All results are shown as mean value ± SD (*n* = 3). Different letters indicate statistical differences (*p* < 0.05) among experimental treatments.

The relative growth rates (RGRs) were significantly affected by pCO_2_ and copper individually and interactively (Figure 1; Table S1). At the lower pCO_2_ level, there were no significant differences between the control and Lcu, while the RGRs significantly decreased from 21.69% d^−1^ (control) to 19.81% d^−1^ (Mcu) and 16.22% d^−1^ (Hcu), respectively. At the higher pCO_2_ level, no significant differences were found at different copper concentrations. Additionally, the RGRs at the higher pCO_2_ level were significantly higher than those at the lower pCO_2_ level under both Mcu and Hcu conditions. However, no significant differences were found between the two pCO_2_ levels in control and Lcu conditions.

### 2.3. Chlorophyll Fluorescence

**Figure 2:**
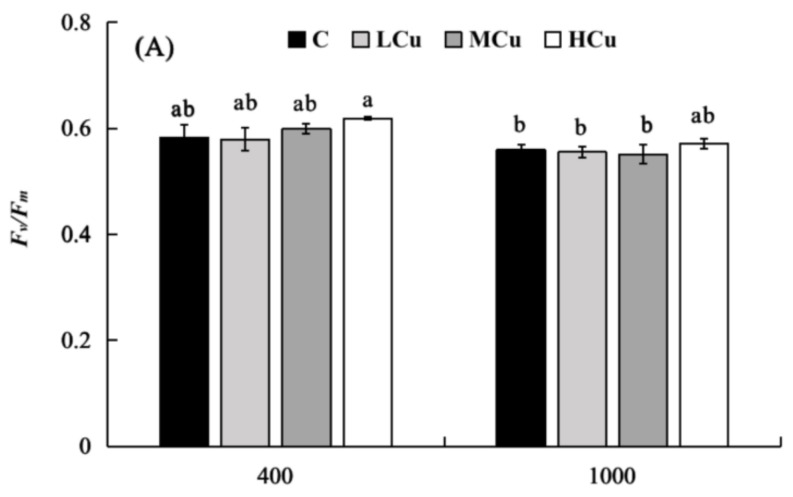

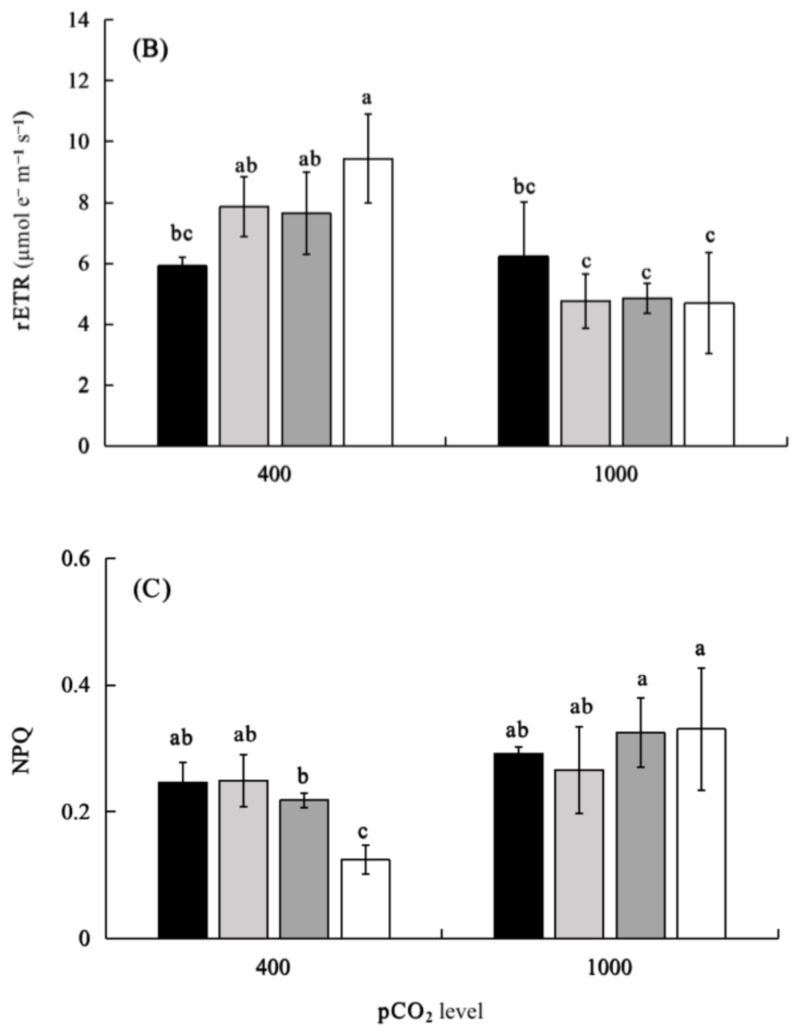
Photosynthetic parameters ((**A**), *F_v_/F_m_*; (**B**), rETR; (**C**), NPQ) of *S. japonica* grown at different pCO_2_ levels and copper concentrations. C (control, natural seawater); LCu (low copper concentration, 0.2 μM); MCu (medium copper concentration, 0.5 μM); HCu (high copper concentration, 0.2 μM). All results are shown as mean value ± SD (*n* = 3). Different letters indicate statistical differences (*p* < 0.05) among experimental treatments.

The *F_v_/F_m_* values were significantly affected by the pCO_2_ level and copper (Figure 2, Table S1). However, there was no significant interaction between pCO_2_ and copper. At the lower pCO_2_ level, the *F_v_/F_m_* values increased with the rise in copper concentration, but the *F_v_/F_m_* values were only significantly increased by 6.24% at Hcu compared to those in the control. In contrast, the *F_v_/F_m_* values were not significantly changed by different copper concentrations at the higher pCO_2_ level. Meanwhile, the higher pCO_2_ concentration significantly decreased the *F_v_/F_m_* values by 8.07% and 7.65% at Mcu and Hcu, respectively, compared to the lower pCO_2_ level. However, no significant differences were found in *F_v_/F_m_* between the two pCO_2_ concentrations in control and Lcu conditions.

The relative electron transport rate (rETR) values were significantly affected by the pCO_2_ level, but they were not significantly influenced by copper (Figure 2, Table S1). A significant interaction between pCO_2_ and copper was detected. At the lower pCO_2_ level, with increasing copper concentration, the rETR values increased by 59.73% at Hcu compared to those in the control. At the higher pCO_2_ level, no significant differences were found in rETR values in any copper treatments. Similarly, there were no significant differences between the two pCO_2_ levels in the control. However, under Lcu, Mcu, and Hcu conditions, the rETR values were significantly decreased by 39.40%, 36.45%, and 50.28%, respectively, at the higher pCO_2_ level compared to those at the lower pCO_2_ level.

The non-photochemical quenching (NPQ) values were significantly affected by the pCO_2_ level, but they were not significantly influenced by copper (Figure 2, Table S1). A significant interaction between pCO_2_ and copper was detected. At the lower pCO_2_ level, with increasing copper concentration, the NPQ values only significantly decreased by 49.71% at Hcu compared to the control. At the higher pCO_2_ level, there were no significant differences at different copper concentrations. Similarly, NPQ values had no significant differences between the two pCO_2_ concentrations in control and Lcu conditions. However, under Mcu and Hcu conditions, the NPQ values at the higher pCO_2_ level were significantly increased by 48.79% and 166.37%, respectively, compared to those at the lower pCO_2_ level.

### 2.4. Pigment Contents

**Figure 3:**
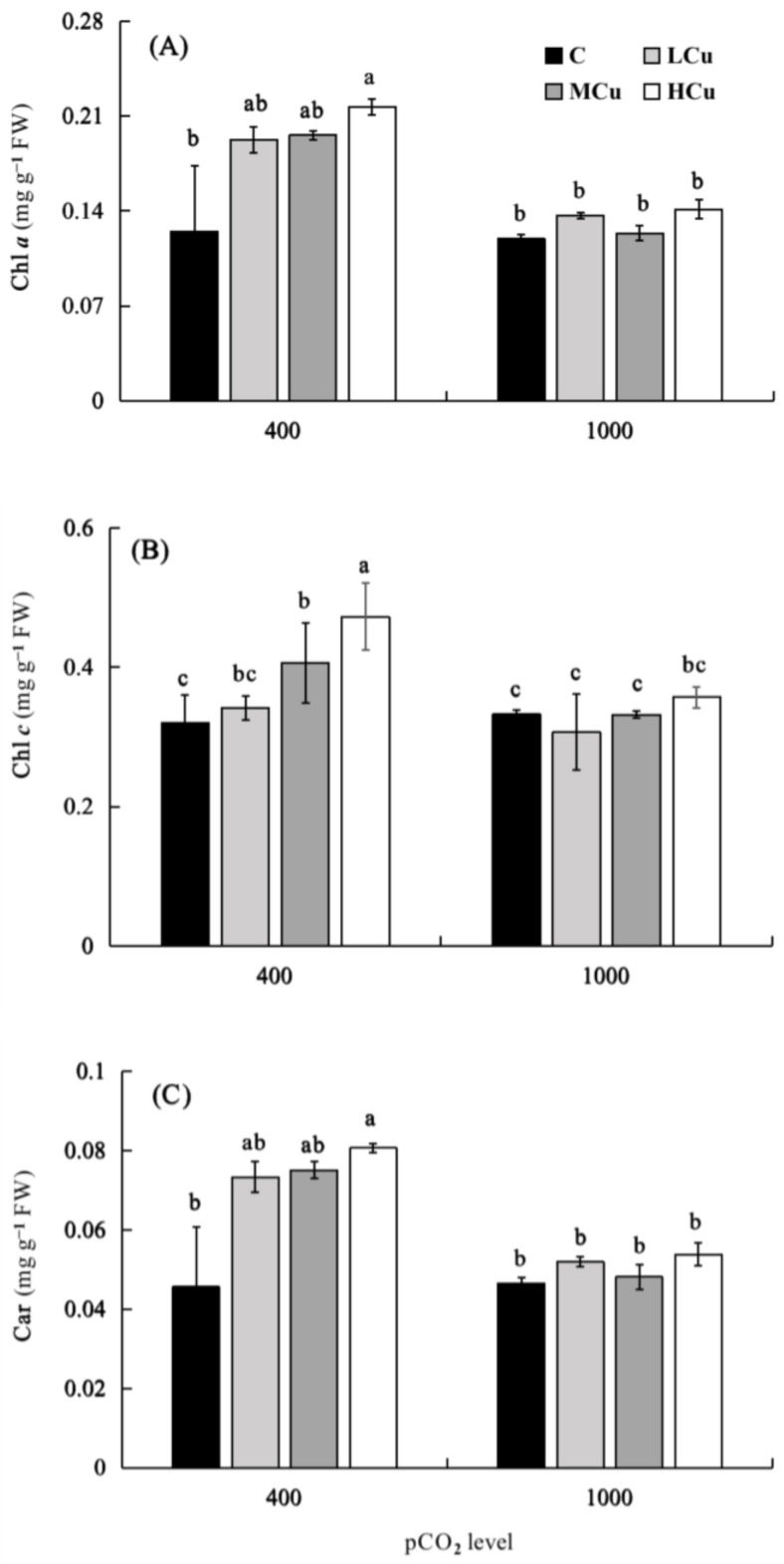
Chlorophyll *a* (**A**), chlorophyll *c* (**B**), and carotenoid content (**C**) of *S. japonica* grown at different pCO_2_ levels and copper concentrations. C (control, natural seawater); LCu (low copper concentration, 0.2 μM); MCu (medium copper concentration, 0.5 μM); HCu (high copper concentration, 0.2 μM). All results are shown as mean value ± SD (*n* = 3). Different letters indicate statistical differences (*p* < 0.05) among experimental treatments.

The contents of chlorophyll *a* (Chl *a*), chlorophyll *c* (Chl *c*), and carotenoid (Car) were significantly affected by pCO_2_ and copper individually and interactively (Figure 3; Table S1). At the lower pCO_2_ level, the contents of Chl *a*, Chl *c*, and Car increased with increasing copper concentrations. Compared with the control, Chl *a* and Car, respectively, were significantly increased by 73.45 and 77.56% at HCu, and Chl *c* was significantly increased by 27.22% and 47.80% at MCu and HCu, respectively. However, at the higher pCO_2_ level, the contents of Chl *a*, Chl *c*, and Car had no significant differences at any of the copper concentrations. Moreover, the contents of Chl *a* and Car at the higher pCO_2_ level significantly decreased by 34.91% and 33.22%, respectively, compared to the lower pCO_2_ level under the HCu condition. At the same time, higher pCO_2_ significantly decreased Chl *c* by 18.34% and 24.41% at Cu and HCu, respectively, compared to the lower pCO_2_ level.

### 2.5. Soluble Carbohydrate Contents

**Figure 4:**
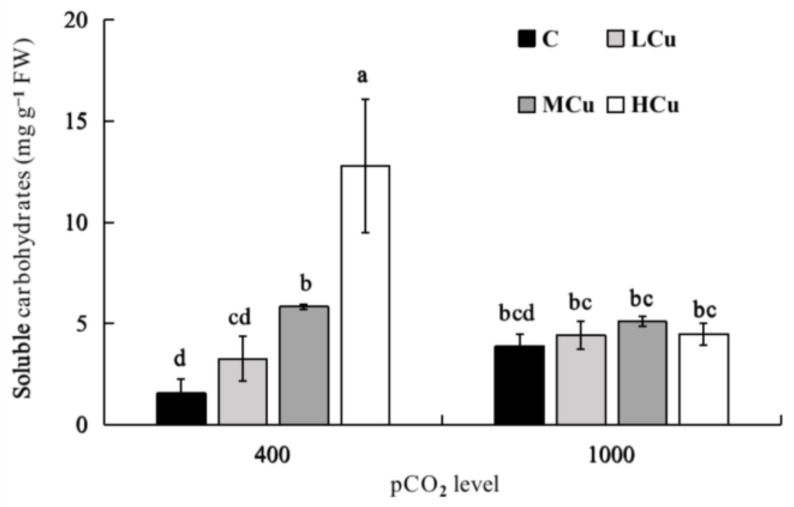
Soluble carbohydrate contents of *S. japonica* grown at different pCO_2_ levels and copper concentrations. C (control, natural seawater); LCu (low copper concentration, 0.2 μM); MCu (medium copper concentration, 0.5 μM); HCu (high copper concentration, 0.2 μM). All results are shown as mean value ± SD (*n* = 3). Different letters indicate statistical differences (*p* < 0.05) among experimental treatments.

The soluble carbohydrate contents were significantly affected by pCO_2_ and copper individually and interactively (Figure 4, Table S1). At the lower pCO_2_ level, with the rise in copper concentration, the soluble carbohydrate contents at MCu and HCu were significantly increased by 273.44% and 718.75% compared to those in the control. However, at the higher pCO_2_ level, the soluble carbohydrate contents did not significantly differ at any of the copper concentrations. Additionally, under the HCu condition, the soluble carbohydrate contents at the higher pCO_2_ level were significantly decreased by 64.89% compared to those at the lower pCO_2_ level.

## 3. Discussion

### 3.1. The Effect of Copper on Growth and Photosynthetic Physiology at Lower pCO_2_ Level

Copper is not only an essential trace element for macroalgae growth but also a highly toxic element at higher concentrations. In this study, the growth of juvenile *S. japonica* sporophytes was progressively reduced in the MCu and HCu treatments at a lower pCO_2_ level. High copper concentrations have been shown to reduce growth in *Gracilariopsis longissima* [1], *Gracilaria lemaneiformis* [40], *Hizikia fusiformis* [41], *P. haitanensis* [10], *Chondrus crispus*, and *Palmaria palmata* [42]. Excess copper in algae may trigger the synthesis of intracellular ROS [41,43] and impair nutrient assimilation [10,41,44], resulting in decreased growth. On the other hand, the harmful effects of copper on algal growth can be attributed to photosynthetic inhibition. However, in the present study, we found a decoupling between reduced growth and increased photosynthetic efficiency, which is consistent with the reports for *Porphyra haitanesis* [45], *Ascophyllum nodosum*, and *Fucus vesiculosus* [46]. The reason may be that under stress conditions, algae reallocate energy from growth to the maintenance of cellular integrity and the accumulation of compounds such as phytochelatins [47], enzymes [48], pigments [12], and other antioxidant molecules [49], thus resisting the adverse effects of copper [1,50]. This result was evidenced by the enhanced pigment contents in HCu and lower-pCO_2_ conditions (Figure 3). Thus, when juvenile sporophytes of *S. japonica* are exposed to higher copper concentrations, the redistribution of energy and disruption in physiological processes may lead to a reduction in algal growth.

In terms of the characteristics relating to chlorophyll fluorescence, excessive copper exposure increased the algal *F_v_*/*F_m_* and rETR but decreased NPQ at the lower pCO_2_ level. A similar response has been shown in *Fucus serratus* under copper exposure [51]. The chlorophyll fluorescence parameter *F_v_*/*F_m_* is an indicator of photoinhibition and a measure of the efficiency of the photosynthetic apparatus of PSII in macroalgae [42]. Given that the toxicity of heavy metals is typically thought to be dose-dependent, exposure to higher copper concentrations or for a longer duration may cause greater damage [42,52]. Hence, the increased *F_v_*/*F_m_* and rETR in this investigation may suggest that the addition of 1 μmol/L copper to the culture may be not adequate to impair the photosynthesis of *S. japonica*. This is supported by research on the brown alga *A. nodosum* [46], which showed that its *F_v_*/*F_m_* and rETR values were not significantly affected at lower concentrations of Cu^2+^ (up to 1 mg/L) but were significantly inhibited at 5 mg/L. In addition, the growth rate of *A. nodosum* was significantly reduced at a 0.1 mg/L copper concentration. As a result, this study’s findings that copper exposure enhanced photosynthesis (*F_v_*/*F_m_* and rETR) and reduced the growth rate reveal that photosynthesis in juvenile *S. japonica* has a higher tolerance for copper toxicity than growth. The higher electron transfer rate in the excess copper condition in this study may also be due to the production of functional Cu-containing proteins involved in photosynthetic electron transport, such as plastocyanin [53,54], as copper participates in important biological processes as an electron carrier in photosynthesis at certain concentrations [55]. The photosynthetic efficiency would also benefit from the increased pigment concentration. At HCu under the lower-pCO_2_ condition, NPQ was decreased, in contrast to the elevated *F_v_*/*F_m_* and rETR. In macroalgae, non-photochemical quenching is a form of photoprotective dissipation of entrapped excitation energy through the activation of the xanthophyll cycle, mostly as heat, preventing damage to the photosynthetic apparatus [56]. The decrease in NPQ under copper exposure may indicate that copper affected the photoprotection system of this alga and caused the xanthophyll pool to shrink or become less robust. Hence, the lower NPQ found in this study may indicate that NPQ, rather than *F_v_*/*F_m_* or rETR, would be the first target of copper toxicity in juvenile sporophytes of *S. japonica*.

Chl *a*, Chl *c*, carotenoid, and soluble carbohydrate contents in juvenile *S. japonica* were increased with rising copper concentrations at the lower pCO_2_ level. Previously, it was noted that *H. fusiformis* [41], *Sargassum cymosum* [4], *U. prolifera* [12], and *Cystoseira tamariscifolia* [57] all had higher levels of photosynthetic pigments. Increased pigments may be crucial in reducing the toxicity of copper, even if photosynthetic pigments are responsible for light-energy absorption in the photosynthesis process of algae [58]. First, increased Chl *a* and Chl *c* at higher copper levels could be an adaptive strategy, completing the pool of chlorophyll to counteract copper toxicity, since magnesium (Mg^2+^) in chlorophyll molecules could replace intercellular Cu^2+^ to decrease the copper toxicity in algae [59]. Secondly, because carotenoids have antioxidative properties [43], and toxic compounds increase the amount of ROS [60], the enhancement of carotenoid content under excess copper conditions in this work may indicate that the alga is subjected to oxidative stress, and carotenoids appear to be an antioxidant substrate used to defend against ROS. Costa et al. [4] and Celis-Plá et al. [57] reported similar responses of photosynthetic pigments, but in these studies, a decoupling phenomenon between the increase in pigments and the decrease in photosynthesis was observed. This contradicts the finding of this study, which found that *S. japonica* was more resistant to excess copper.

In terms of soluble carbohydrates as photosynthetic products, active photosynthesis may lead to their synthesis by increasing *F_v_*/*F_m_*, rETR, and pigment contents. Furthermore, according to some studies, the carbohydrates excreted by algae can regulate the concentration of copper in the environment and restrict metal uptake by forming complexes [8,61,62]. Consequently, in order to release more carbohydrates into seawater, the alga may tend to stimulate the production of photosynthetic organic compounds, establishing an important barrier to protect cells from the harmful effects of excess copper.

### 3.2. The Effects of Elevated pCO_2_ Level on Growth and Photosynthetic Physiology in Ambient Copper Condition

In contrast to the effects of copper, the elevated pCO_2_ level did not significantly change the growth rate, chlorophyll fluorescence parameters, or biochemical composition in the absence of copper. Kang and Chung [36] also reported that higher pCO_2_ concentrations had no effect on the growth or chlorophyll fluorescence parameters of *S. japonica*. Similar phenomena have been observed in other brown algae, including *S. fusiforme* [7] and *Sargassum horneri* [63]. To overcome the carbon limitation in seawater, most macroalgae can use gaseous CO_2_ and the ionic form of HCO_3_^−^ to drive photosynthesis via carbon-concentrating mechanisms (CCMs). Some macroalgae whose photosynthesis is carbon-limited at current pCO_2_ levels would benefit from OA because CO_2_ and HCO_3_^−^ concentrations in seawater increased markedly with OA. The lack of changes in the photosynthetic apparatus under the elevated-pCO_2_ condition in this study may indicate that algal photosynthesis is nearing saturation at the ambient inorganic carbon level [6], and thus, no more carbon was fixed by photosynthesis or contributed to growth.

### 3.3. The Effects of Higher Copper on Growth and Photosynthetic Physiology at Elevated pCO_2_ Level

When exposed to higher copper concentrations (0.2~1 µM), no significant differences in any of the parameters were observed at the elevated pCO_2_ level. This indicates that OA weakened the sensitivity of the growth and photosynthetic physiology of *S. japonica* to excess copper under interactive conditions. In this study, when the copper concentration was increased to 0.5 µM, elevated pCO_2_ offset the negative effect of copper on growth at the lower pCO_2_ level. Furthermore, at 1 µM copper concentration, the alleviated effect was more noticeable. These findings indicate that the effects of excess copper on this alga at the ambient pCO_2_ level could be mitigated by OA, and the combined effects of elevated pCO_2_ and copper levels could be found when the copper concentration was increased to 0.5 µM.

Previous studies suggest that the lower sensitivity of the growth and photosynthetic physiology of juvenile *S. japonica* to excess copper at higher pCO_2_ levels may be related to the competition between H^+^ and Cu^2+^ [12,64,65]. When H^+^ in seawater increased, Cu^2+^ would be competitively excluded from binding to ligands at the cell surface [64,65], reducing the toxicity of copper. This phenomenon was also found by Gao et al. [12], who noted that the growth of *U. prolifera* was less negatively affected by 0.5 μmol/L copper at 1000 ppmv compared to 390 ppmv. Moreover, increased concentrations of other free trace metal ions (Me^2+^) at low pH may be another reason. At low pH, free trace metals became more soluble and less likely to bind to organic matter due to proton competition; thus, the concentrations of the other free Me^2+^ were increased in water [66]. However, low-toxicity free Me^2+^, such as Mn^2+^, Zn^2+^, and Fe^3+^, may be able to outcompete the highly toxic Cu^2+^ for binding ligands at the cell surface, lowering copper uptake and toxicity in algae [66]. Because of the poorer absorption of copper in algae and the competition between Cu^2+^ and H^+^ and/or other free Me^2+^ for binding biological ligands at the cell surface when pH decreased, we hypothesized that OA protected the thalli of *S. japonica* against copper toxicity. However, further studies are required to assess the accumulated volume of copper in the alga under different pCO_2_ and copper treatments.

In conclusion, this work evaluated the interaction between pCO_2_ and the copper concentration on the growth and physiological characteristics of the juvenile sporophyte of *S. japonica*. At an ambient pCO_2_ level, the growth of *S. japonica* was inhibited by higher copper concentrations (0.5~1 µM), but its photosynthesis (*F_v_/F_m_* and rETR), pigment contents (Chl *a*, Chl *c*, and Car), and SC contents were all improved. This may suggest that when this alga responds to copper stress, its growth and photosynthesis become decoupled. It is interesting to note that high pCO_2_ reduced the toxic effects of copper on the growth of the kelp. Likewise, OA also reduced the beneficial impacts of excess copper on the photosynthetic physiology and biochemical compositions at low pCO_2_. As a result, our findings show that *S. japonica* was more resistant to excess copper at elevated pCO_2_ levels, suggesting that it would be well adapted to future oceanic conditions and have the potential to be used in the treatment of wastewater. This finding may also indicate that, throughout the artificial seedling process, higher pCO_2_ levels in seawater will help juvenile *S. japonica* resist the harmful effects of heavy metals. However, in order to obtain a more comprehensive view of the combined effects of OA and copper pollution on the growth and physiology of *S. japonica*, further research on the response of different developmental stages of this alga is needed.

## 4. Materials and Methods

### 4.1. Sample Collection and Maintenance

Juvenile sporophytes of *S. japonica* were collected from cultivated populations in Lianjiang, Fujian, China (26°07′ N, 120°17′ E), in December 2021. These samples were put into a cooling box filled with seawater and transported quickly to the laboratory within 2 h. Selected healthy individuals (approximately 5 cm in length) were rinsed with filtered natural seawater to remove epiphytic organisms and then pre-cultured in an intelligent illumination incubator (GXZ-380B, Jiangnan Instrument Factory, Ningbo, China). In order to prevent nutrient depletion, filtered sterile seawater enriched with 25% PESI [67] was used to culture the samples. Natural seawater was obtained from the coastal region of Pingtan, Fuzhou. A preliminary analysis of seawater using an atomic absorption spectrophotometer (AA6300C, SHIMADZU, Japan) showed that the copper concentration was 0.01 μmol/L. The temperature was set to 9 °C with a 12 h:12 h (light/dark) photoperiod of 60 μmol photons m^−2^ s^−1^.

After 3 days, they were cultured in side-arm flasks containing 500 mL of 25% PESI-enriched seawater. Cultures were maintained under four different copper regimes (control, C; 0.2 μM, LCu; 0.5 μM, MCu; 1 μM, HCu) and two pCO_2_ levels (400 and 1000 ppmv) to investigate the combined effects of OA and copper. The choice of copper concentration was based on the report by Li et al. [68]. Natural seawater without the addition of CuSO_4_·5H_2_O (Sigma Aldrich, Steinhelm, Germany) was regarded as the control, and the copper content in the natural seawater was 0.01 μmol/L. The two pCO_2_ levels were maintained in two separate CO_2_ incubators and automatically regulated by controlling the flows of ambient air and pure CO_2_ gas. The algal stocking density was about 0.3 g fresh weight. The cultures were conducted in three replicates for each experiment and lasted for 9 days and bubbled with ambient or CO_2_-enriched air at a rate of 0.4 L min^−1^ to make the thalli roll up and down. The culture medium was changed every 3 days. The temperature and light conditions were consistent with the preincubation conditions described above. The TA and pH of the culture medium were determined after 24 h aeration before the algae were cultured. TA samples were immediately collected in acid-washed glass bottles without empty space, preserved with a few drops of saturated HgCl_2_ solution, and stored at room temperature until analysis. The pH (NBS scale) was measured using a pH meter (Orion STAR A211; Thermo Scientific), and the TA was calculated by titration. The seawater carbonate system parameters were calculated using the CO2SYS software [65,69], according to known parameters, such as temperature, salinity, TA, and pH.

### 4.2. Measurement of Growth Rates

Before and after the experiment, the fresh weights of all juvenile sporophytes were measured. The samples were dried on paper towels until no changes in biomass were detectable. The RGR (% day^−1^) of each replicate was estimated as follows:

(1) $$\mathrm{RGR}=100\times \left({\mathrm{lnW}}_{t}-{\mathrm{lnW}}_{0}\right)/t\;\;$$

where W_0_ is the initial fresh weight, and W_t_ is the final fresh weight after t days of culture.

### 4.3. Measurement of Chlorophyll Fluorescence

After the culture experiment, the chlorophyll fluorescence parameters of juvenile sporophytes were measured using a pulse modulation fluorometer (Imaging-PAM, Walz, Germany). Before determination, the samples were kept in the dark for 15 min. The basal fluorescence (F_0_) was obtained at low light, and the maximum fluorescence (F_m_) was measured by the saturation pulse method with an intensity of 4000 µmol m^−2^ s^−1^. *F_v_/F_m_* was calculated using the equation *F_v_*/*F_m_* = (F_m_ − F_0_)/F_m_. The rETR and NPQ were detected under actinic light in our experiments after 15 min of dark adaptation. The rETR = ΔF/F_m_ × PAR × 0.5, where ΔF/F_m_ represents the effective PSII quantum yield, PAR is the light irradiance, and 0.5 refers to the fraction of incident PAR absorbed by chlorophyll associated with PSII. NPQ was calculated as follows: NPQ = (F_m_ − F_m_’)/F_m_’, where F_m_ represents the maximum fluorescence after 15 min of darkness adaptation, and F_m_’ represents the maximum fluorescence yield under actinic light.

### 4.4. Measurements of Pigments

Approximately 0.1 g (fresh weight) of samples from each culture condition was used for the extraction of Chl *a* and Car. These samples were placed in 10 mL of methanol for 24 h in the dark, and the absorption of the supernatant was measured at 750, 665, 652, and 510 nm with an ultraviolet absorption spectrophotometer (U-2900, HITACHI, Tokyo, Japan). The contents of Chl *a* and Car were estimated according to [70,71].

Approximately 0.2 g (fresh weight) of samples in each treatment was used for the extraction of Chl *c*. These samples were placed in 2 mL of dimethyl sulfoxide for 5 min, and the absorption of the supernatant was detected at 665, 631, and 582 nm using an ultraviolet absorption spectrophotometer. Next, the same samples were placed in 3 mL of acetone for 2 h. The supernatant was transferred into a 10 mL tube, and 1 mL of methanol and 1 mL of distilled water were then added. The absorbance of the supernatant was read at 664, 631, and 581 nm. The concentrations of Chl *c* were estimated according to [72]. Pigment contents were determined as mg g^−1^ FW.

### 4.5. Measurement of Soluble Carbohydrates

About 0.1 g of the fresh alga was homogenized in 2 mL of distilled water and diluted to 10 mL. The homogenate was centrifuged at 1400× *g* for 5 min at 4 °C. After centrifugation, 1 mL of the supernatant was transferred to a glass tube and diluted to 2 mL with distilled water, and then 8 mL of anthrone reagent was added. The mixture was immersed in boiling water for 10 min. After cooling at room temperature, the absorbance value of the sample at 620 nm was measured. The contents of soluble carbohydrates were calculated according to [73] and expressed as mg g^−1^ FW.

### 4.6. Statistical Analysis

All data are expressed as the mean ± SD (*n* = 3). Prior to the analysis, tests for the normal distribution (Shapiro–Wilk, *p* > 0.05) and homogeneity (Levene’s test, *p* > 0.05) of variance were conducted. A two-way analysis of variance (ANOVA) was used to test the combined effects of the pCO_2_ level and copper on the seawater carbonate parameters, RGR, *F_v_/F_m_*, rETR, NPQ, Chl *a*, Chl *c*, Car, and soluble carbohydrates. A Duncan test was used to analyze the significance level of the factors (*p* < 0.05). All data analyses were performed using SPSS 22.0 software.

## Figures and Tables

**Table 1:** Carbonate parameters of seawater at different pCO_2_ levels and copper concentrations. C (control, natural seawater); LCu (low copper concentration, 0.2 μM); MCu (medium copper concentration, 0.5 μM); HCu (high copper concentration, 0.2 μM). Data are shown as mean value ± SD (*n* = 3). Different letters indicate statistical differences (*p* < 0.05) among experimental treatments.

Copper	pCO_2_ (ppmv)	pH	TA (μmol L^−1^)	DIC (μmol kg^−1^)	CO_3_^2−^ (μmol kg^−1^)	HCO_3_^−^ (μmol kg^−1^)	CO_2_ (μmol kg^−1^)
Control	400	7.90 ± 0.02 ^a^	2230 ± 18 ^a^	2132 ± 24 ^a^	84.8 ± 3.1 ^a^	2020.9 ± 25.6 ^a^	26.6 ± 1.6 ^a^
	1000	7.53 ± 0.04 ^c^	2223 ± 8 ^a^	2232 ± 7 ^b^	38.5 ± 3.7 ^c^	2128.2 ± 4.8 ^b^	65.4 ± 6.8 ^c^
LCu	400	7.89 ± 0.02 ^a^	2234 ± 15 ^a^	2137 ± 21 ^a^	84.4 ± 3.1 ^a^	2026.0 ± 22.3 ^a^	26.9 ± 1.6 ^a^
	1000	7.58 ± 0.01 ^b^	2228 ± 18 ^a^	2223 ± 20 ^b^	42.9 ± 0.7 ^bc^	2122.3 ± 18.7 ^b^	57.9 ± 1.8 ^b^
Mcu	400	7.87 ± 0.03 ^a^	2227 ± 21 ^a^	2138 ± 16 ^a^	80.1 ± 5.0 ^a^	2029.0 ± 14.3 ^a^	28.4 ± 1.7 ^a^
	1000	7.58 ± 0.03 ^b^	2232 ± 4 ^a^	2228 ± 6 ^b^	42.8 ± 3.0 ^bc^	2126.5 ± 4.7 ^b^	58.6 ± 4.2 ^b^
Hcu	400	7.88 ± 0.02 ^a^	2224 ± 7 ^a^	2131 ± 8 ^a^	81.1 ± 2.6 ^a^	2023.9 ± 8.9 ^a^	27.9 ± 1.1 ^a^
	1000	7.60 ± 0.03 ^b^	2216 ± 13 ^a^	2205 ± 17 ^b^	44.7 ± 2.9 ^b^	2105.6 ± 16.0 ^b^	55.0 ± 4.1 ^b^

## Data Availability

The data of this study are available from the corresponding author upon reasonable request.

## Supplementary Materials

The following supporting information can be downloaded at https://www.mdpi.com/article/10.3390/plants12051140/s1: Table S1: Analysis of variance (two-way ANOVA) examining the effects of pCO_2_ level and copper condition on RGR, *F_v_*/*F_m_*, rETR, NPQ, Chl *a*, Chl *c*, Car, and soluble carbohydrates of juvenile sporophytes of *Saccharina japonica*.
