# Supplementary material for: Response of Juvenile Saccharina japonica to the Combined Stressors of Elevated pCO2 and Excess Copper
Source: Plants (Basel). 2023 Mar 2;12(5):1140. doi: 10.3390/plants12051140 (PMC10005686; doi:10.3390/plants12051140)
Supplement: Supplementary file 1 [file plants-12-01140-s001.zip › plants-2171931-supplementary.pdf]

Supplementary data for the article:

Article

Response of Juvenile *Saccharina japonica* to the Combined Stressors of Elevated pCO_2 and Excess Copper

Wenze Zhang [1,2,3], Lianghua He [1,2], Jiangqi Pan [1,2], Yuhong Zhou [1,2], Ruxiang Ge [1,2], Sufang Li [4], Yunyun Shi [1,2], Xinhua Chen [1,2,*], and Yaoyao Chu [1,2,*]

[1] College of Marine Sciences, Fujian Agriculture and Forestry University, Fuzhou 350002, China
[2] Key Laboratory of Marine Biotechnology of Fujian Province, Institute of Oceanology, Fujian Agriculture and Forestry University, Fuzhou 350002, China
[3] Department of Aquaculture and Aquatic Sciences, Kunsan National University, Gunsan 54150, South Korea
[4] Laboratoire Génie des Procédés et Matériaux (LGPM), CentraleSupélec, Université Paris-Saclay, 91190 Gif-sur-Yvette, France
* Correspondence: chenxinhua@tio.org.cn; chuyaoyao@fafu.edu.cn

Table S1. Analysis of variance (two-way ANOVA) examining the effects of pCO_2 level and copper condition on RGR, F_v/F_m, rETR, NPQ, Chl *a*, Chl *c*, Car and soluble carbohydrates of juvenile sporophytes of *Saccharina japonica*.

Factors	df	F	*P*
**RGR**			
pCO_2	1	66.853	< 0.001
Copper	3	12.913	< 0.001
pCO_2 × Copper	3	15.001	< 0.001
F_v/F_m			

pCO_2	1	32.396	< 0.001
Copper	3	3.829	0.031
pCO_2 × Copper	3	1.309	0.306
**rETR**			
pCO_2	1	26.661	< 0.001
Copper	3	0.786	0.519
pCO_2 × Copper	3	4.464	0.018
**NPQ**			
pCO_2	1	20.616	< 0.001
Copper	3	0.972	0.430
pCO_2 × Copper	3	4.169	0.023
**Chl *a***			
pCO_2	1	51.247	< 0.001
Copper	3	10.890	< 0.001
pCO_2 × Copper	3	4.966	0.013
**Chl *c***			
pCO_2	1	66.853	< 0.001
Copper	3	12.913	< 0.001
pCO_2 × Copper	3	15.001	< 0.001
**Car**			
pCO_2	1	60.415	< 0.001
Copper	3	14.894	< 0.001
pCO_2 × Copper	3	7.666	0.002
**Soluble carbohydrates**			
pCO_2	1	6.749	0.019
Copper	3	23.195	< 0.001
pCO_2 × Copper	3	19.967	< 0.001
